# Effect of the Matrix Melt Flow Index and Fillers on Mechanical Properties of Polypropylene-Based Composites

**DOI:** 10.3390/ma15217568

**Published:** 2022-10-28

**Authors:** Harri Junaedi, Muneer Baig, Abdulsattar Dawood, Essam Albahkali, Abdulhakim Almajid

**Affiliations:** 1 Department of Engineering Management, College of Engineering, Prince Sultan University, P.O. Box 66833, Riyadh 11586, Saudi Arabia; 2 Saudi Arabian Basic Industries Corporation (SABIC), Plastics Applications Development Center, Riyadh 12373, Saudi Arabia; 3 Department of Mechanical Engineering, College of Engineering, King Saud University, P.O. Box 800, Riyadh 11421, Saudi Arabia

**Keywords:** polypropylene, short carbon fiber, titanium dioxide, nanocomposite, mechanical properties

## Abstract

In this work, mechanical properties of reinforced polypropylene composites were studied. PP in particulates shape with two different melt flow indexes (MFI) was used, i.e., 3 and 23 g/10 min, namely PP3 and PP23, respectively. Three different materials, namely TiO_2_ nanoparticle (nTiO_2_, spherical, 0D), micro-size short carbon fiber (SCF, fiber, 1D), and graphite nanoplatelet (GNP, sheet, 2D), were used as reinforcements/fillers. PP and fillers (in the desired composition) were first pre-mixed by a mechanical mixer. The mixture was then fed to a co-rotating twin-screw extruder for melt-compounding, followed by injection molding to fabricate testing samples. The microstructure and fracture surface of the composites were observed by a scanning electron microscope (SEM). Additionally, tensile, flexural, impact, and hardness tests were conducted to evaluate their mechanical properties. The SEM images stipulate that PP23 had better adhesion and dispersion with the fillers. The results from the SEM images support the mechanical testing results. PP23 composites exhibited more significant improvement in mechanical properties in comparison to PP3. At 5 wt. % filler loading, PP/GNP composite exhibited a greater improvement in mechanical properties compared with two other composites, which are PP/SCF and PP/nTiO_2_ composites for both PPs.

## 1. Introduction

The primary factor that drives the composite industry is the great demand for lightweight materials. Composites are replacing metallic (aluminum and steel) parts in many industries. The increasing demand for lightweight materials provides a boost for composite industries. The demand for advanced materials continues to fuel the composite industry for better composites [1]. The exceptional properties of PP composites, including being lightweight and posessing high strength and recyclability, made them great candidates for automotive applications. The automotive industry is expected to have the highest growth in utilizing PP composites in the next decade due to the high load-to-weight ratio and strength. The corrosion resistance of PP composites has also fueled the growth in the PP composite market [2].

Different reinforcing fillers are being used to improve the mechanical and physical properties of polymer-based composites. Fillers are solid particulate materials usually used in polymers to enhance their properties [3]. Different types of filler material and their loading levels in the polymer may affect the properties of the polymer composite differently [4]. Carbon-fiber-reinforced composites gained popularity in the industry due to their low weight and high strength. The high cost of processing carbon fiber compared to glass fiber limited the utilization of carbon fiber in advanced industries [1]. The use of fibers and nanomaterials-reinforced thermoplastic composites existed for many years. Meanwhile, graphene or graphite nano-platelet (GNP) composites gained popularity in the last decade. The growth in the graphene market is fuelled by the demand for renewable and lightweight materials.

SCF-reinforced polymer composites have been studied since the 1970s. Nevertheless, until today, PP/SCF composites are still interesting research topics [5,6,7]. Meanwhile, the polymer nanocomposite has become a research trend and one of the research focuses in the polymer field for the past 15 years. PP/GNP [8,9,10,11] and PP/TiO_2_ nanoparticles [12,13,14,15] composites have been investigated extensively by many researchers. The addition of a small amount of GNP to PP was reported to improve the mechanical properties of the composite significantly [8]. GNP and TiO_2_ nanoparticles were also reported can be functionalized as anti-ultraviolet [16,17,18] and flame retardant [19,20,21].

The properties of composite polymers are not only affected by fillers but also depend on the base polymer and the interfacial bonding between the base polymer and the filler [22]. The melt flow index (MFI), in general, characterizes the rheological aspect of polymers, which is known as melt viscosity [23]. MFI value and viscosity have an inverse relation, wherein the higher MFI value corresponds to lower melt viscosity [24]. The MFI of the matrix influences the ability to mix between the matrix and fillers. Polymers with a low melt viscosity (high MFI) present better wetting and homogeneous distributions of the filler. These characteristics are expected to enhance the interaction between polymer and fillers and, moreover, increase the performance of a composite. Meanwhile, high melt viscosity (low MFI) polymers could lead to poor wetting and distribution. They could weaken the interfacial bonding between the polymer matrix and filler and lower the mechanical properties of composites [25,26].

In this study, different filler materials, namely TiO_2_ nanoparticles (nTiO_2_), short carbon fiber (SCF), and graphite nano-platelet (GNP), were incorporated into the PP matrix with two different melt flow indexes to produce PP-based composites. The fillers were selected based on their dimensions, such as 0D, 1D, and 2D. Incorporating these PPs and fillers creates a broad spectrum of composite materials to be characterized and studied. This paper emphasizes studying the effect of different types of PPs and fillers on the mechanical properties of composites.

## 2. Materials and Methods

### 2.1. Materials

**Figure 1:**
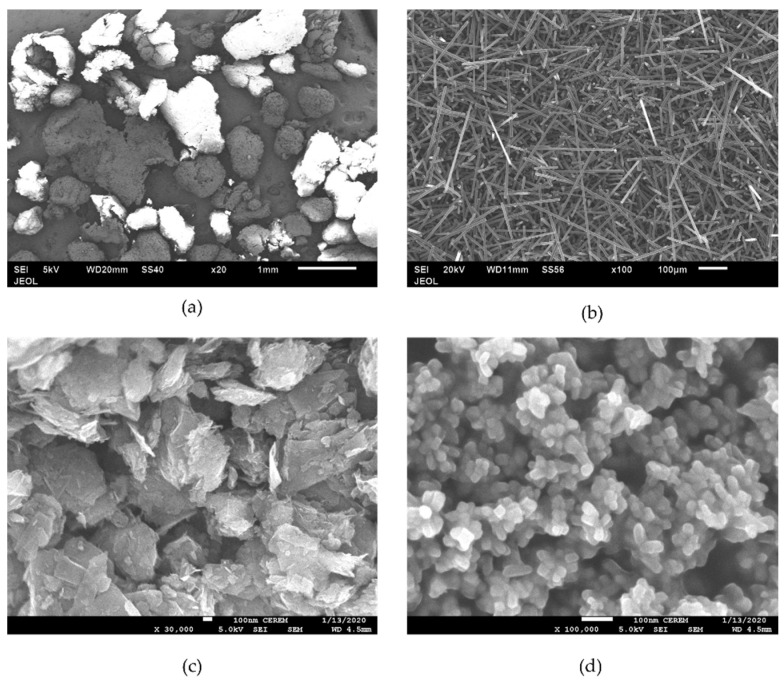
SEM images of raw materials (**a**) PP23, (**b**) SCF, (**c**) GNP, and (**d**) nTiO_2_.

The raw materials used were the isotactic polypropylene (iPP) polymer as the matrix and SCF, GNP, and nTiO_2_ as the fillers (Figure 1). Two different types PPs with different MFI were used. Table 1 shows the properties of the PPs. The first PP was PP500 grade with an MFI of 3 g/10 min (PP3) and the second PP was PP511A grade with an MFI of 23 g/10 min (PP23). MFI was measured at a temperature of 230 °C and a load of 2.16 kg. Both PPs were in the form of particulates and were supplied by SABIC. The milled SCF with 7–9 µm diameter and 150 µm average length (with carbon content above 94%) was purchased from Asbury Carbon Inc. The GNP was Nano307 grade obtained from Asbury Carbon Inc. The thickness of the GNP was about 3 nm and a diameter of less than 1 µm. A 30 nm average diameter nTiO_2_ rutile grade was used and purchased from US Research Nanomaterials, Inc.

### 2.2. Processing of Composites

**Figure 2:**
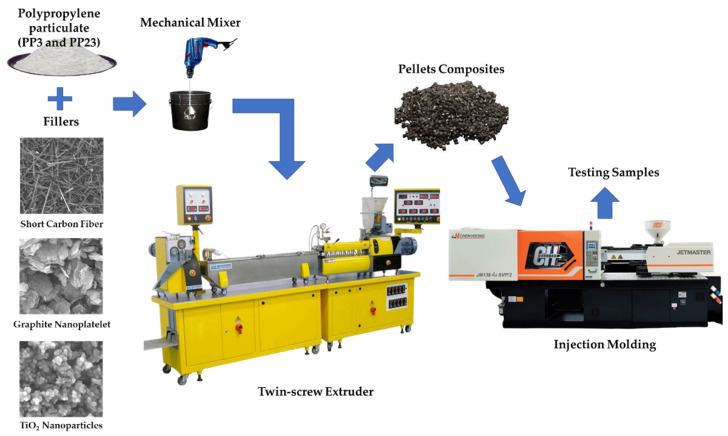
Processing steps schematic of the composites.

The PP-based composites used in this study were prepared by combining varying percentages of SCF, GNP, and nTiO_2_ with the PP matrix. The percentage of SCF in the composites varied from 0 to 20 wt. %. Meanwhile, for nanofillers, the concentration varied between 0 and 5 wt. %. Table 2 presents the detailed compositions of the composites used in this study. Initially, the PP polymer was dry-mixed with the desired percentage of fillers by a mechanical mixer. The mixed mixture was then fed and melt-mixed in a twin-screw extruder. The temperature at which the mixture was processed was selected within the range of 150–210 °C to obtain composite pellets. The injection molding machine was used to produce the tensile test specimens from the composite pellets. The barrel temperature of the injection molding process was selected within the range of 180–220 °C, while the nozzle temperature was 230 °C. The schematic of composites manufacturing process can be seen in Figure 2.

### 2.3. Characterization of Composites

#### 2.3.1. Microstructure

The observation of the microstructure of the composites was performed on the test sample. It was performed using a Scanning Electron Microscope (JEOL JSM 6610LV). The surface of the sample was initially coated with electroplating before SEM observation.

#### 2.3.2. Tensile Test

**Figure 3:**
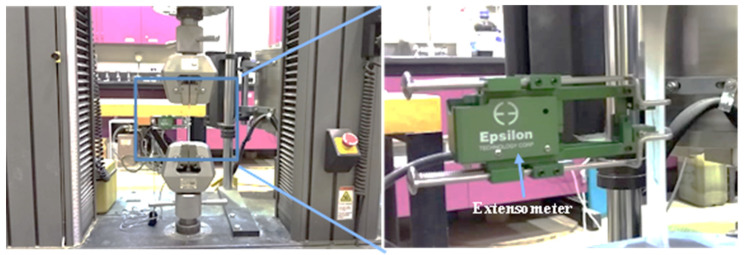
Tensile testing.

Uniaxial tensile tests were carried out per the ASTM D638-14 standard to obtain the mechanical properties of the composites [27]. The sample dimension was based on Type I of ASTM D638. Room temperature tests were performed using INSTRON 3385H with a 5 mm/min loading speed. An extensometer was attached to the samples to measure accurate strain. The ultimate tensile strength (UTS), tensile modulus, and strain at break were calculated from the stress–strain curve. Three experiments were performed at each composition to obtain statistically significant data. The tensile test’s setup can be observed in Figure 3.

#### 2.3.3. Flexural Test

**Figure 4:**
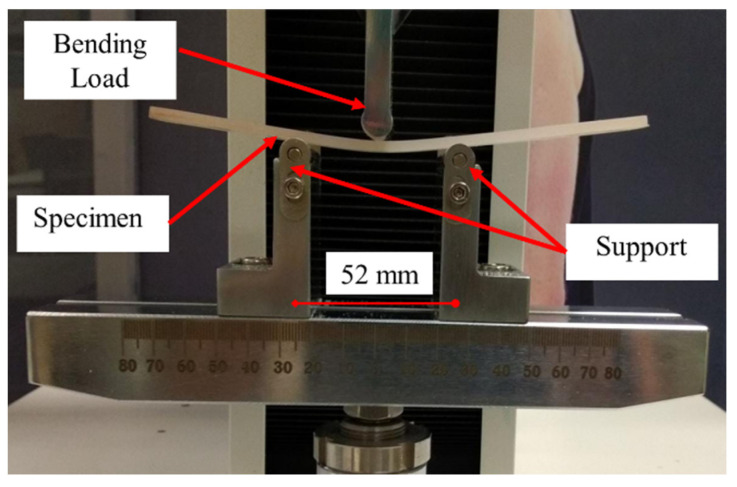
Three-point bending testing.

Flexural tests were performed according to ASTM D790-10 [28] on an INSTRON 3385H with a three-point bending standard attachment (Figure 4). Three experiments were carried out on each composition. The cross-head speed was adjusted to 1.5 mm/min, with a span support distance set at 52 mm. Flexural modulus and strength were calculated from the experiments.

#### 2.3.4. Impact Test

**Figure 5:**
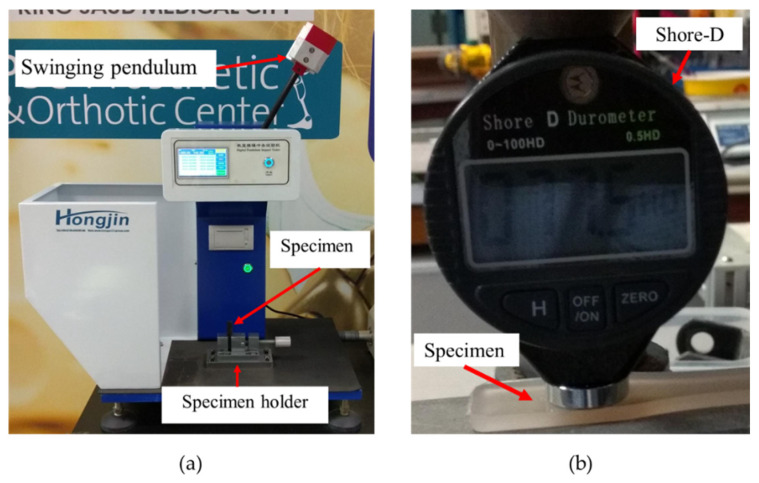
(**a**) Izod impact and (**b**) hardness shore-D testing.

The Izod impact tests were conducted as per ASTM D256-10 [29] with notched samples to study the notch impact’s toughness (kJ/m^2^). An Izod impact test machine with an impact speed of 3.5 m/s and maximum impact energy of 5.5 J was used (Figure 5a). Three tests were conducted for each composition.

#### 2.3.5. Hardness Test

Hardness tests were conducted on the composites by Durometer according to ASTM D2240-15 [30] type D (Shore-D, Figure 5b). Five measurements were performed for every sample. Readings were taken 1 s after the needle touched the samples.

## 3. Results and Discussion

### 3.1. Microstructures

**Figure 6:**
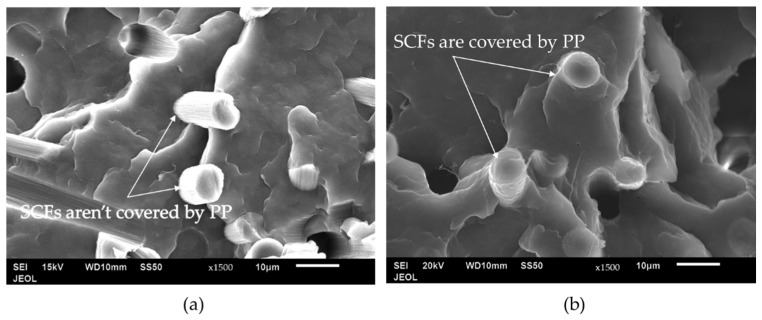
SEM images of 20 wt. % of SCF-reinforced Polypropylene (PP/20SCF) of (**a**) PP3 and (**b**) PP23.

Figure 6 shows the SEM images of PP/20SCF composites with two different types of PP. It shows that PP23 has a better interface compared to PP3. Additionally, in the PP23/20SCF composite, the fibers appear to be covered by the PP matrix. However, the figure for the PP3/20SCF composite shows clean fiber surfaces, implying that the PP is not completely attached to the SCF’s surface. Since PP23 was processed with identical processing conditions as PP3, it can be concluded that the PP with high MFI (PP23) can have better wettability relative to the SCF in comparison to PP with a lower MFI (PP3) [31].

**Figure 7:**
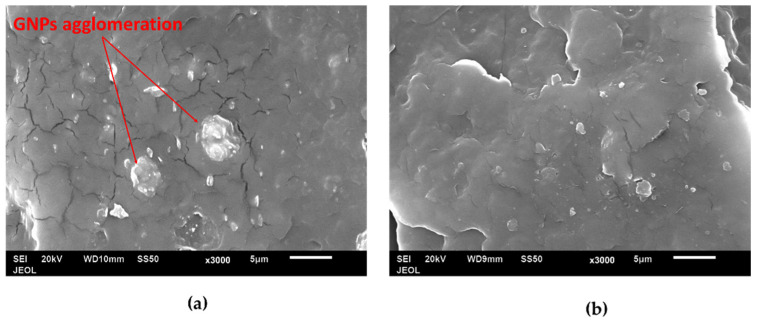
SEM images of 5 wt. % of GNP of PP/5GNP composites of (**a**) PP3 and (**b**) PP23.

SEM images of PP/GNP composites are shown in Figure 7. These composites were composed of 5 wt. % GNP. It is observed from Figure 7b that the PP23 composite has a better dispersion compared to PP3. The agglomeration of GNP was observed in PP3 composites, as shown in Figure 7a.

**Figure 8:**
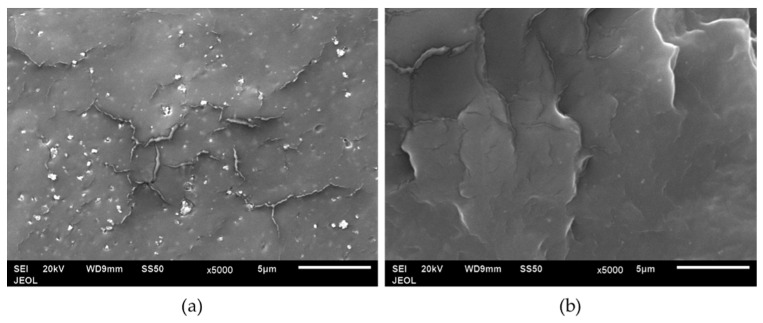
SEM images of 5 wt. % of nTiO_2_ of PP/5nTiO_2_ composites of (**a**) PP3 and (**b**) PP23.

Figure 8 reveals the microstructure of the PP/nTiO_2_ composites. nTiO_2_ tends to agglomerate in the PP3 composite at 5 wt. % nTiO_2_ loading (Figure 8a). Meanwhile, in Figure 8b, PP23 shows a better dispersion of nTiO_2_ when compared to PP3. Thus, it can be inferred that PP with higher MFI performs better in the filler’s dispersion [25].

### 3.2. Tensile Test

**Figure 9:**
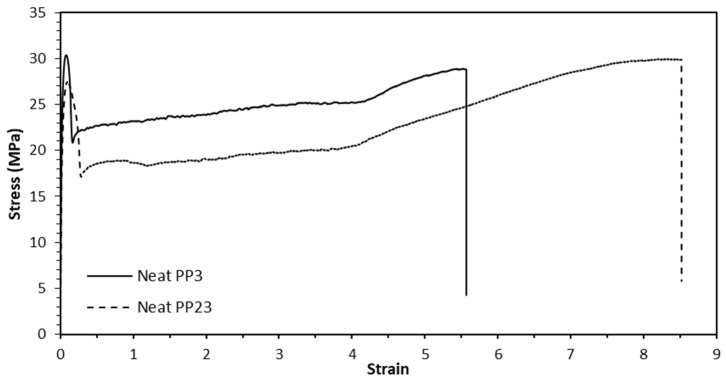
Typical stress-strain curves for neat PP3 and PP23.

Figure 9 shows the typical stress–strain curves for neat PP3 and PP23. The average mechanical properties from the tensile test of the two different PPs are presented in Table 3. From the figure, it is observed that PP3 has a higher tensile modulus and UTS compared to PP23, but PP23 has a higher strain to break compared to PP3. Even though PP3 has higher UTS than PP23, PP23 has higher stress at fracture.

**Figure 10:**
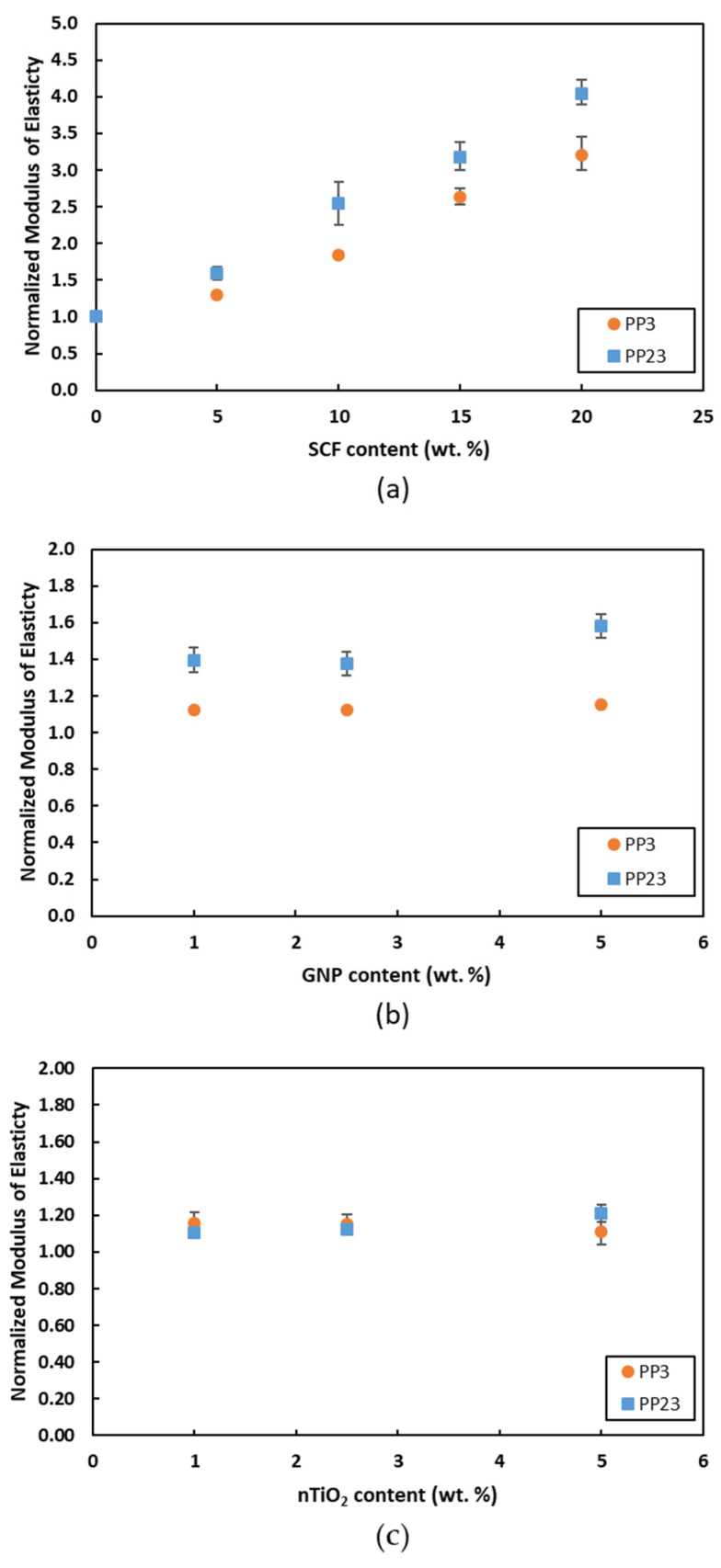
Normalized tensile modulus of (**a**) PP/SCF, (**b**) PP/GNP, and (**c**) PP/nTiO_2_ composites.

Figure 10 shows the normalized modulus of elasticity of PP composites. Figure 10a shows the normalized modulus of elasticity of PP/SCF composites. It is shown that at 20 wt. % of SCF loading, the tensile modulus of PP23/20SCF increases by 306% compared to 222% increases for the PP3/20SCF. Figure 10b shows the normalized modulus of elasticity of PP23/GNP composites. PP23/GNP composites have a slightly higher increase on the average tensile modulus compared to PP3/GNP, and the differences are more pronounced at 5 wt. % of GNP. Figure 10c shows that the tensile modulus of PP23/nTiO_2_ composites has a slightly higher increase of up to 21% on average at 5 wt. % loading. However, PP3/nTiO_2_ exhibited a 16% maximum increase at 1 wt. % loading and then the tensile modulus started to drop. The higher increase in the tensile modulus of PP23/SCF composites signifies that PP23 has better interfacial bonding with the SCF compared to PP3. It is known that interfacial bonding can also be affected by wettability. The difference between PP3 and PP23 is in the melt flow index of the polymer. The polymer with a higher MFI at the same processing temperature has better wettability [31].

**Figure 11:**
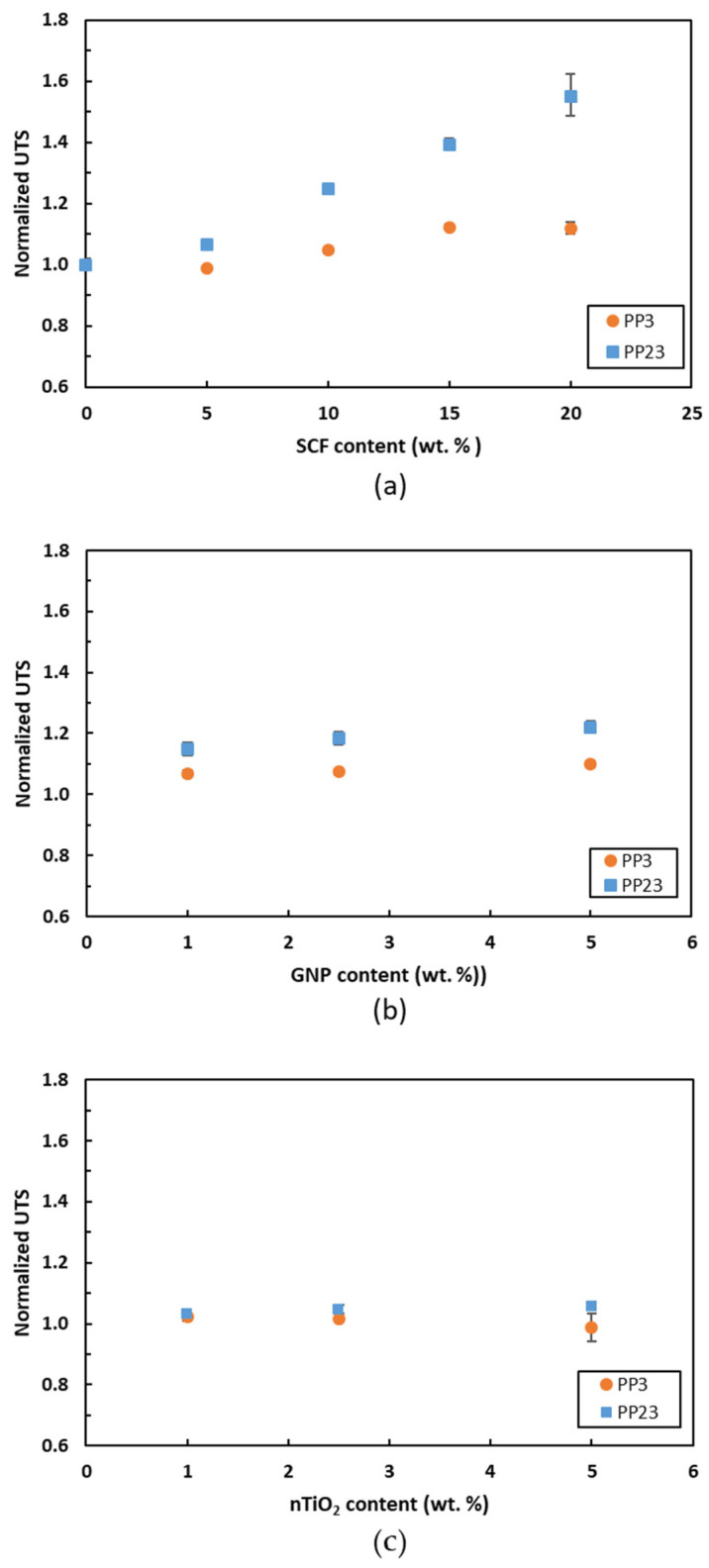
Normalized UTS of (**a**) PP/SCF, (**b**) PP/GNP, and (**c**) PP/nTiO_2_ composites.

Figure 11 shows the UTS of PP composites. Figure 11a presents the UTS of PP/SCF composites. The UTS of PP23/20SCF increases by 55.4%, compared to the PP23/20SCF, which is only 12%. The higher increase in UTS of PP23/SCF is also attributed to the superior interfacial bonding with the SCF compared to PP3. Good interfacial bonding can also be affected by good wettability [32]. Figure 11b shows the UTS of PP/GNP composites. The UTS of PP23/GNP also shows a higher increase compared to PP3. At 5 wt. % of GNP, PP23/5GNP increases by up to 22%. However, the UTS of PP23/5GNP increases by up to 10%. The UTS of PP23/nTiO_2_ also shows an increase while PP3/nTiO_2_ shows a decrease. At 5 wt. % of nTiO_2_, PP23/5nTiO_2_ increases by almost 6%; meanwhile, the PP3/5nTiO_2_ decreases by 1.4%, as shown in Figure 11c. This phenomenon could be attributed to the poor dispersion of the filler on the matrix. The agglomeration or aggregation of nanofillers is responsible for the drop of the tensile modulus or/and UTS [33]. PP with a higher MFI could lead to better dispersion of the fillers [25]. A comparison among the three fillers showed that GNP provided better performance in the tensile modulus and UTS for loading up to 5 wt. % for both PPs. A higher specific surface area of the GNP than the other fillers is responsible for this phenomenon.

**Figure 12:**
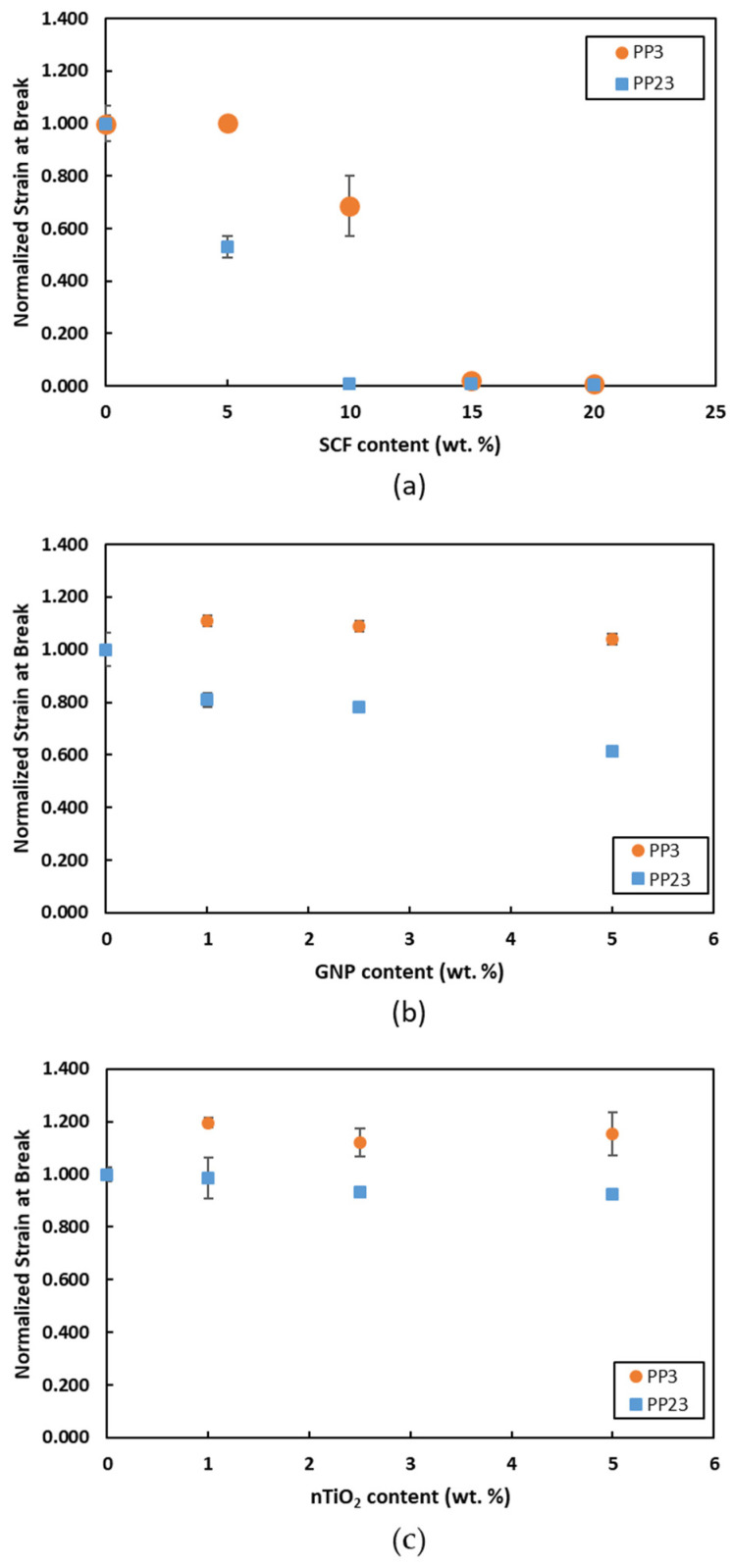
Normalized strain at break of (**a**) PP/SCF, (**b**) PP/GNP, and (**c**) PP/nTiO_2_ composites.

Figure 12 shows the strain at break comparison of PP/SCF, PP/GNP, and PP/nTiO_2_ composites of two different PP. From Figure 12a, the PP23/SCF composite’s strain at break drops faster than the PP3/SCF composite. For PP23/SCF composite, the strain at break value drops to 1% strain of the neat PP23 at 10 wt. % SCF loading. Meanwhile, PP3/10SCF still has high ductility, which is 69% of neat PP3 strain. Both PPs show a sudden drop in the strain at break value at different SCF loadings. Higher interfacial bonding between PP and SCF inhibits the movement of the PP chain molecule, which also leads to lower strains at break. The sudden drop of strain at break at certain loadings has been discussed elsewhere [6]. Figure 12b shows the strain at break for PP/GNP composites. The PP23/GNP strain at break drops slightly while PP3/GNP composites show some increases compared to neat PP3. PP23/GNP strain at break increased to 576% from 553% at 5 wt. % GNP loading; meanwhile, PP3/GNP dropped from 845% to 520% at 5 wt. % GNP loading. It can be inferred that PP23 also has better interfacial bonding with GNP compared to PP3. Figure 12c shows the strain at break for PP/nTiO_2_ composites. It is shown that for the PP3/nTiO_2_ composite, the strain at break value increases to 638% from 553% at 5 wt. % nTiO_2_ loading; meanwhile, PP23/nTiO_2_ dropped from 845% to 781% at 5 wt. % nTiO_2_ loading. Similarly to the response of the other two fillers, PP23 also has better interfacial bonding with nTiO_2_ compared to PP3.

### 3.3. Flexural Test

**Figure 13:**
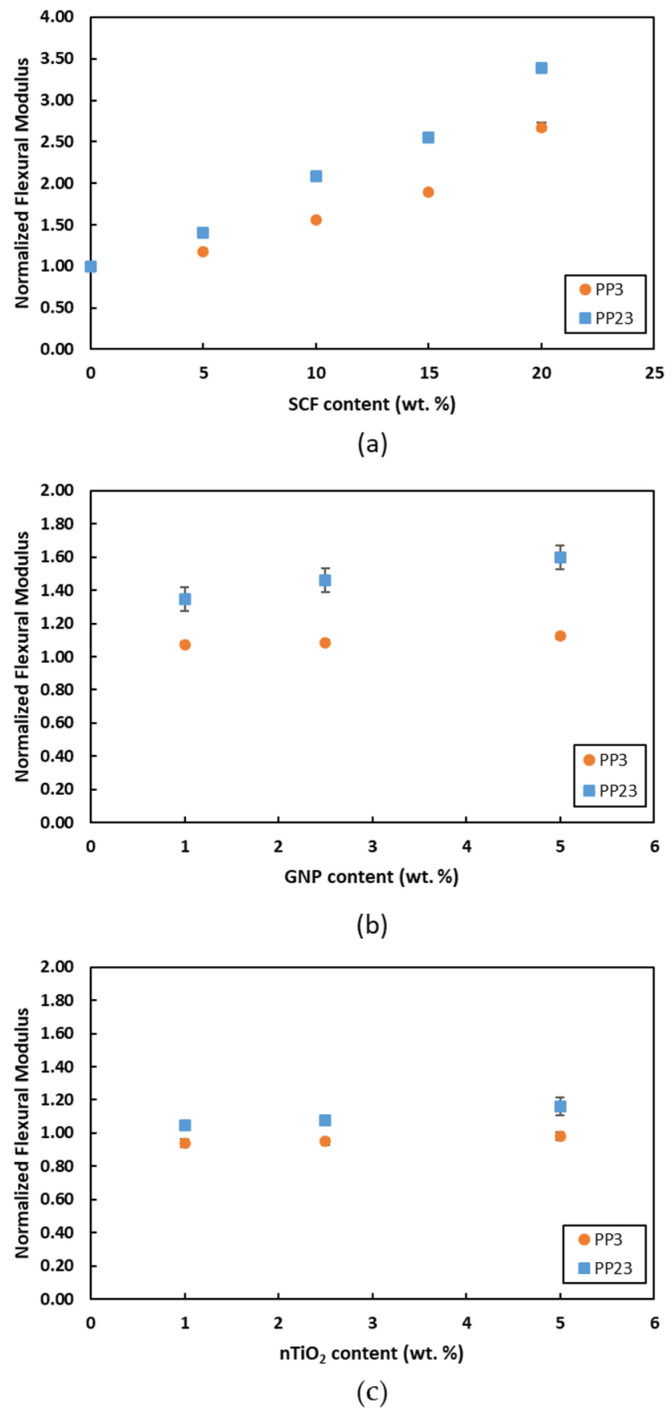
Normalized flexural modulus of (**a**) PP/SCF, (**b**) PP/GNP, and (**c**) PP/nTiO_2_ composites.

**Figure 14:**
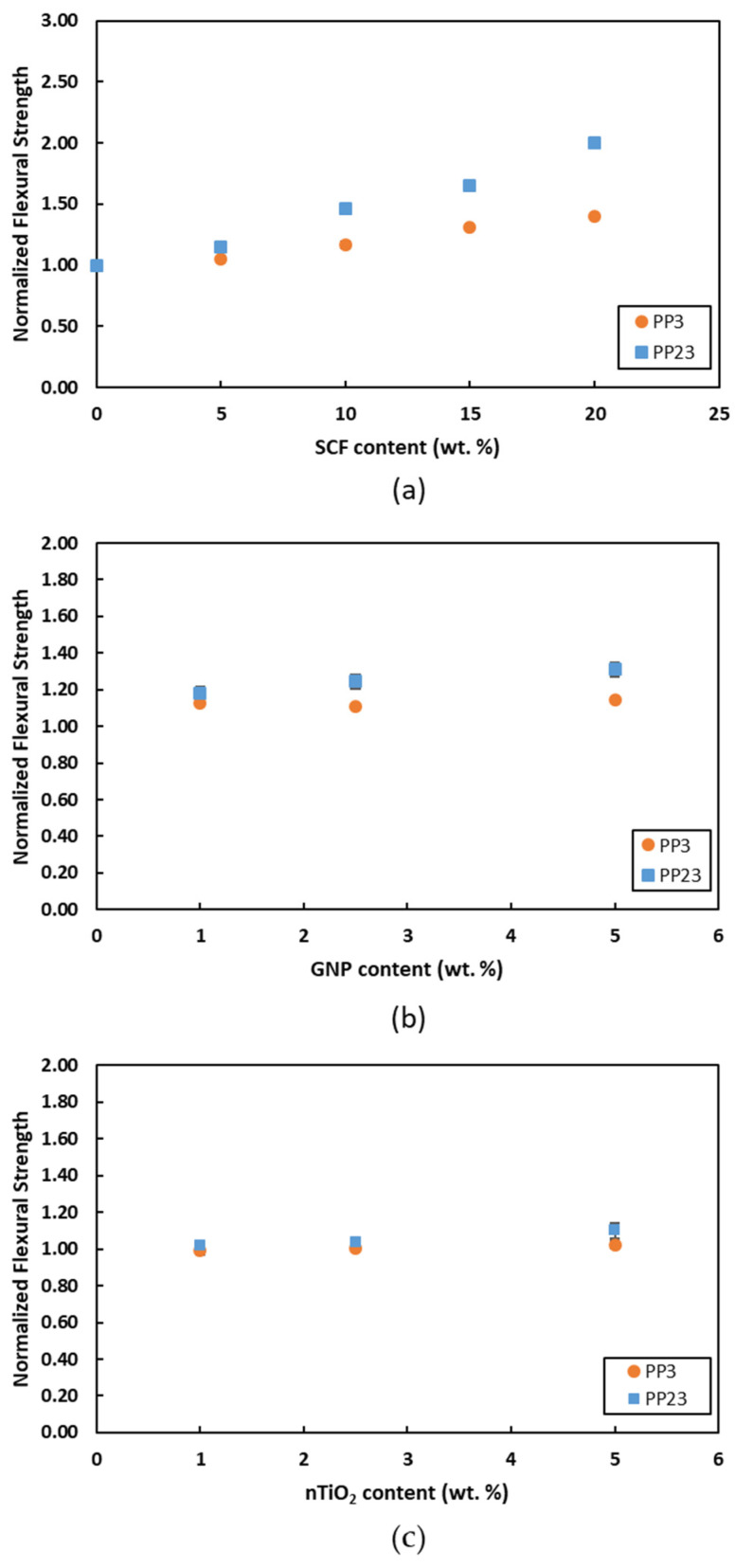
Normalized flexural strength of (**a**) PP/SCF, (**b**) PP/GNP, and (**c**) PP/nTiO_2_ composites.

Figure 13 and Figure 14 show the comparison of flexural modulus and flexural strength, respectively. In general, an improvement in the flexural modulus and flexural strength can be observed for both PPs with the increase in filler loading. However, PP23 composites exhibit higher improvements in properties when compared to PP3 composites. Figure 13a and Figure 14a show that PP23/SCF composites exhibit higher increases in the flexural modulus value and flexural strength value compared to PP3/SCF composites. The maximum increase in the flexural modulus and flexural strength of the PP23/20SCF composite was 240% and 100%, respectively, compared to an increase of 170% and 40%, respectively, for the PP3/20SCF composite. The better interfacial bonding between PP23 and SCF is a major factor responsible for such behaviors. Figure 13b and Figure 14b show the flexural modulus and flexural strength of PP/GNP composites. The figures show that PP23/GNP composites exhibit a higher improvement in the flexural modulus value and flexural strength value when compared to PP3/GNP composites. Figure 13c and Figure 14c show the flexural modulus and flexural strength of PP/nTiO_2_ composites. From the figures, it is observed that PP23/nTiO_2_ composites exhibit a higher improvement in flexural modulus and flexural strength compared to PP3/nTiO_2_ composites. Kim et al. [34] also reported similar results for PP/Cotton composite. The tensile and flexural strength of PP/Cotton composite with a higher MFI exhibited higher improvements compared to the lower MFI due to the better wettability. From the observation, it can be inferred that other than wettability, PP23 has a better ability to disperse nanofillers, as mentioned in the previous section. A comparison of the three fillers showed that GNP provided better performance in the flexural modulus and strength for loading up to 5 wt. % for both PPs.

### 3.4. Impact Test

**Figure 15:**
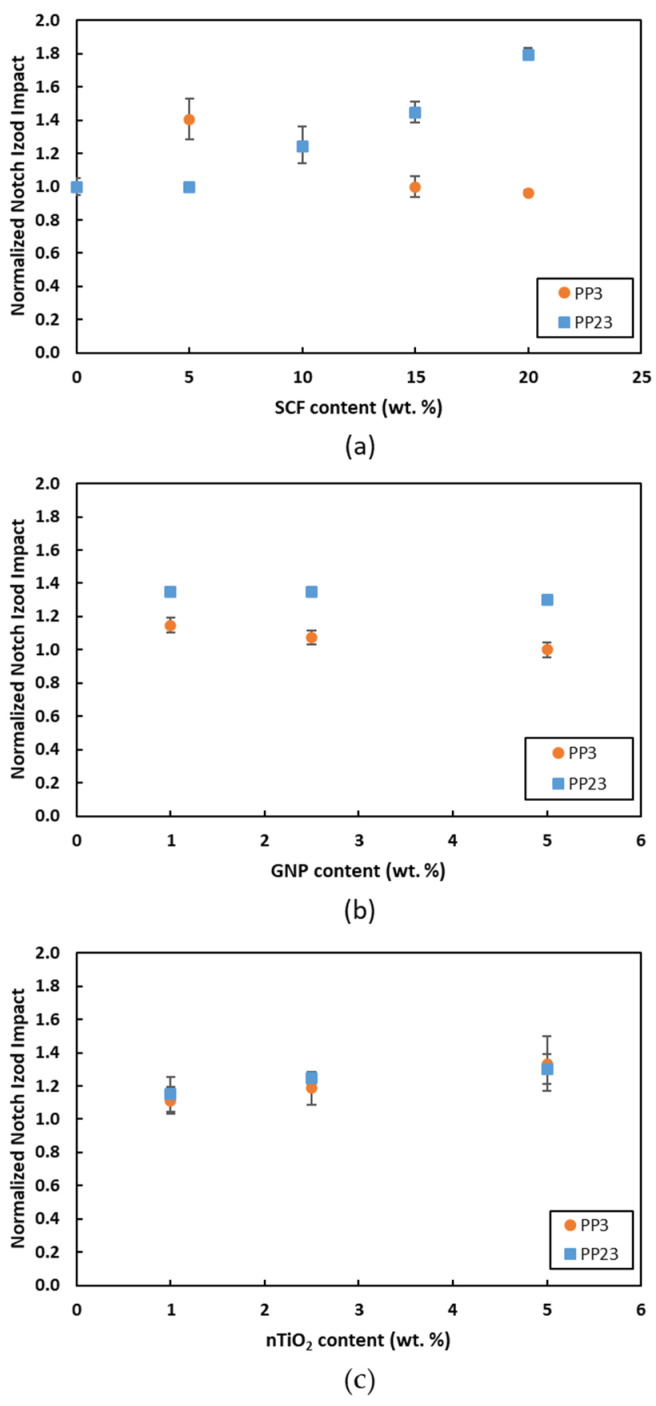
Normalized notch Izod impact toughness of (**a**) PP/SCF, (**b**) PP/GNP, and (**c**) PP/nTiO_2_ composites.

Figure 15 compares the normalized notch Izod impact toughness for PP3 and PP23 composites with different fillers. The impact toughness of PP23/SCF composites shows an increase by the increase in the wt. % loading of SCF, as shown in Figure 15a. Meanwhile, PP3/SCF composites show an increase at 5 wt. % loading but then dropped again and almost stayed steady by the increase in wt. % of SCF. This steady impact toughness of PP3/SCF composites could be attributed to crack deflection, low interfacial bonding, and fibers’ orientation to the fracture surface. Due to the presence of SCF in the composite, the crack deflection would increase the impact toughness value of the composite. On the other side, low interfacial bonding between SCF and PP and SCF oriented to align to the fracture’s surface may affect the toughness values in the composite. For PP23, the synergy between good interfacial bonding and crack deflection increased the notch impact’s toughness values. The notched impact toughness value increased with the increase in wt. % of SCF loading. In addition, fiber fracture and pull-out during the sample fracture are also responsible for improving impact toughness [35].

Figure 15b compares the normalized notch Izod impact toughness for PP3/GNP and PP23/GNP composites. Up to 5 wt. % loading of GNP, the PP23/GNP shows a maximum increase at 1 wt. % loading and then starts to drop as the GNP loading increases. However, the impact toughness value was still higher when compared to the impact toughness value of the neat PP. It shows that PP23 has a better interfacial bonding with GNP than PP3. An improvement in the impact toughness values is attributed to the bridging effect of GNP [8]. Meanwhile, the drop in the impact toughness of PP3/GNP by the further increase in GNP is due to the increase in the agglomeration of GNPs and GNP aligned to the fracture surface [36].

Figure 15c compares the normalized notch Izod impact toughness for PP23/nTiO_2_ and PP23/nTiO_2_ composites. Both composites, PP3/nTiO_2_ and PP23/nTiO_2_, show an improvement in impact toughness by the increase in wt. % of nTiO_2_ up to 5 wt. % loadings. The presence of the spherical shape of filler may produce crack blunting during crack propagation, which is responsible for the improvement of the impact toughness [12,37].

### 3.5. Hardness Test

**Figure 16:**
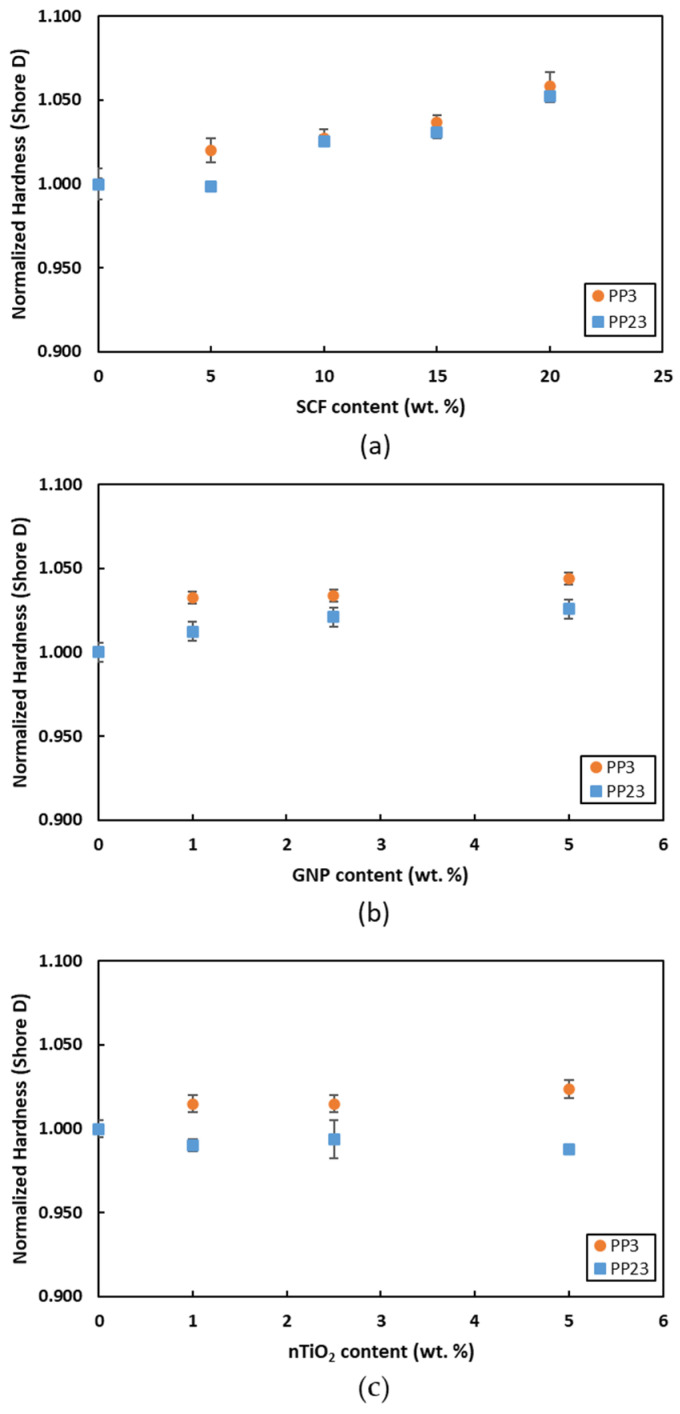
Normalized hardness (Shore-D) of (**a**) PP/SCF, (**b**) PP/GNP, and (**c**) PP/nTiO_2_ composites.

Figure 16 shows the variation in the normalized hardness values of the composites with the varying percentages of the filler materials. For all composites studied in this work, in general, PP3 composites exhibit slightly higher hardness compared to PP23 composites. The presence of fillers hinders the movement of the polymer’s molecular chains. The PP3, which has a higher molecular weight (lower MFI), compared to PP23 (higher MFI) can gain a higher increase in hardness with the addition of the filler. The increase in hardness is more evident with the addition of nanofillers. For 5 wt. % loading, GNP gained a higher hardness increase, followed by nTiO_2_ and SCF. The nanofillers have a higher specific surface area compared to SCF.

## 4. Conclusions

The effect of polypropylenes and fillers types on the mechanical properties of PP-based composites was studied. PP with MFI 23 (PP23)-based composites exhibited a higher tensile modulus, UTS, flexural modulus, flexural strength, and notch impact toughness compared to the PP with MFI 3 (PP3)-based composite. Meanwhile, the hardness of PP3 composites exhibits a slightly higher increase compared to PP23. The microstructures reveal that the PP23 could provide better interfacial bonding and dispersion to the fillers. The composite with the GNP filler exhibits higher enhancements on mechanical properties compared to SCF and nTiO_2_ with up to 5 wt. % loading.

## Figures and Tables

**Table 1:** Physical properties of the polypropylenes.

Polypropylene Type	Name Tag	MFI (g/10 min) @2.16 kg and 230 °C	Density (g/cm^3^)
PP500	PP3	3	0.900
PP511A	PP23	23	0.900

**Table 2:** Composites compositions.

Name Tag	wt. % SCF	wt. % GNP	wt. % nTiO_2_
Neat PP	-	-	-
PP/5SCF	5	-	-
PP/10SCF	10	-	-
PP/15SCF	15	-	-
PP/20SCF	20	-	-
PP/1GNP	-	1	-
PP/2.5GNP	-	2.5	-
PP/5GNP	-	5	-
PP/1nTiO_2_	-	-	1
PP/2.5nTiO_2_	-	-	2.5
PP/5nTiO_2_	-	-	5

**Table 3:** Mechanical properties of the neat polypropylenes.

Type	Tensile Modulus (MPa)	UTS (MPa)	Strain at Break (%)	Stress at Fracture (MPa)
PP3	1643 ± 45	30 ± 0.6	553 ± 38.5	28.7 ± 0.3
PP23	1358 ± 35	27.6 ± 0.3	845 ± 26.1	30.2 ± 0.7

## Data Availability

The data presented in this study are available upon request from the corresponding author.
